# Reconfigurable nonlinear losses of nanomaterial covered waveguides

**DOI:** 10.1515/nanoph-2023-0563

**Published:** 2023-10-23

**Authors:** Ayvaz Davletkhanov, Aram Mkrtchyan, Alexey Bunkov, Dmitry Chermoshentsev, Mikhail Shashkov, Daniil Ilatovskii, Dmitry Krasnikov, Albert Nasibulin, Yuriy Gladush

**Affiliations:** Skolkovo Institute of Science and Technology, Moscow 121205, Russia; Russian Quantum Center, Skolkovo, Moscow 121205, Russia; Boreskov Institute of Catalysis SB RAS, Novosibirsk 630090, Russia

**Keywords:** saturable absorption, carbon nanotubes, optical limiting, optical waveguides

## Abstract

Optical waveguides covered with thin films, which transmittance can be controlled by external action, are widely used in various applications from optical modulators to saturable absorbers. It is natural to suggest that the losses through such a waveguide will be proportional to the absorption coefficient of the covering material. In this letter, we demonstrate that under certain conditions, this simple assumption fails. Instead, we observe that the reduction of the material loss of the film can lead to an increase in the propagation losses through the waveguide. For this, we use a side polished fiber covered with a single-walled carbon nanotube thin film whose absorption can be attenuated either by a short pulse illumination (due to absorption saturation) or with electrochemical gating. For the films thicker than 50 nm, we observe saturable absorption to turn into optical limiting with nonmonotonic dependence on the incident power. With a numerical simulation, we identify that this nontrivial behavior comes from mode reshaping due to changes in the absorption coefficient of the covering film. We demonstrate the applicability of the observed effect by fabricating the device which nonlinear optical response can be controllably switched between saturable absorbing and optical limiting. Finally, we utilize an analytical approach to predict the required parameters and corresponding nontrivial shapes of the nonlinear absorbance curves. These results provide new perspectives for engineering complex reconfigurable nonlinear optical responses and transmittance dependences of nanomaterial covered waveguides.

## 1 Introduction

Covering waveguides with nanomaterials can bring new functionality to the otherwise passive optical elements. Examples include graphene and related 2D materials, carbon nanotubes, vanadium dioxide, topologic insulators, and many others used in integrated photonics for modulators, optical switches, photodetectors, and nanoheaters [1–10]. Another important platform utilizes optical fibers when nanomaterial film covers either a side-polished fiber (SPF) or a tapered fiber to produce sensors [], modulators [18–24], polarizers [22, 25–29], or saturable absorbers [30–35]. The efficiency of these devices directly depends on the light and material interaction strength, which is defined by the overlap integral of the covering material and the evanescent tail of the waveguide mode. Several approaches have been proposed to increase the overlap integral by adjusting the waveguide geometry and the material coating technique [36–40]. For example, an overlayer with a higher refractive index pulls the mode toward covered surface, which can significantly increase the overlap integral [27, 28, 36, 41]. The variation of the optical parameters of the covering material affects the shape of the mode that enables to control the overlap integral, i.e., the interaction strength. Many of the mentioned above applications, like optical switches and amplitude modulators, optical limiters, and saturable absorbers, rely on the change of the imaginary part of the refractive index, while the variation of its real part remains small. The imaginary part variation can also change the overlap integral and consequently the losses through the waveguide.

In this work, we demonstrate theoretically and experimentally that a continuous reduction of the imaginary part of the refractive index of the covering material not necessarily leads to a proportional decrease in the losses through the waveguide but can even cause an increase in the waveguide absorbance. With a help of numerical simulations, we show that this effect can be attributed to the mode reshaping and change in the overlap integral value. For experimental demonstration, we use a practically important system of a polymer-free single-walled carbon nanotube (SWCNT) thin film covering the SPF, which is used for the mode-locking of fiber lasers [42]. We apply two experimental methods to decrease the material absorption: the resonant absorption saturation and electrochemical gating. In both cases, we find that it leads to the absorbance increase through the waveguide for sufficiently thick film in accordance with numerical simulations. Then, we combine these two approaches to demonstrate a device possessing reconfigurable nonlinear loss that can switch between saturable and induced absorption. Finally, we show that this effect can be resembled by a simplified analytical approach that allows us to predict the conditions, under which it can be observed with other covering materials.

## 2 Results and discussion

**Figure 1:**
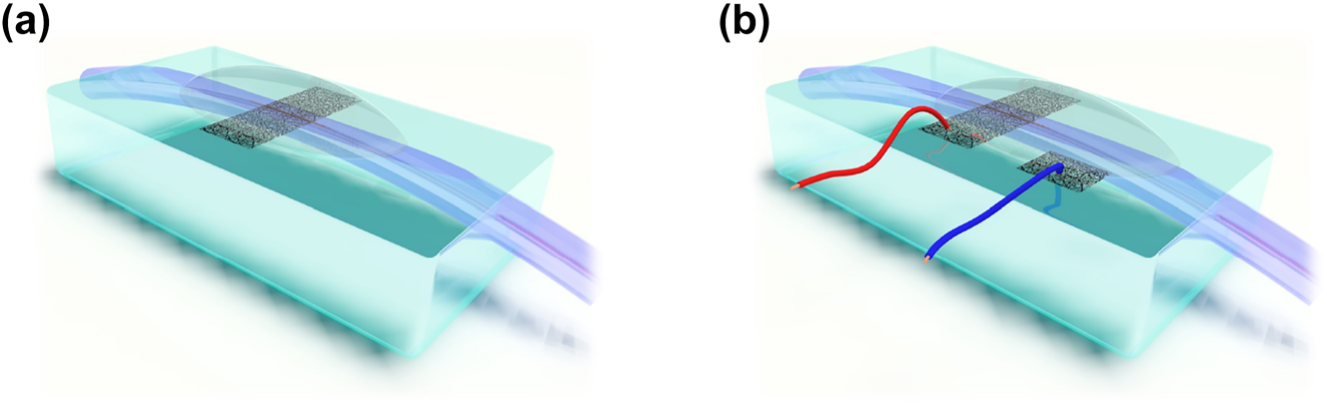
Graphical illustrations of the optical devices under investigation: (a) the SPF with a thin film of SWCNTs covered by a liquid overlayer and (b) the ionic cell to modify the absorption of the carbon nanotube film by electrochemical gating.

We investigate the SPF with a thin film of SWCNTs covered by a liquid overlayer (Figure 1a). The details of the sample preparation can be found in Materials and Methods section. In brief, for SWCNTs, we implement an aerosol floating catalyst synthesis method that allows to obtain a thin film of pure uniform randomly oriented carbon nanotube network with a required thickness without any liquid chemistry steps [43, 44]. The mean diameter of the SWCNTs is tuned during the synthesis to 1.4 nm [45], corresponding to *S*_11_ interband transition at 1.55 μm, which matched with the wavelength of the erbium-doped fiber laser (see the absorbance spectrum of carbon nanotubes in Figure S1 in Supplementary Material). We synthesized a series of SWCNT films with various thicknesses in the range from 10 to 120 nm, which were controlled using AFM (see Figure S2), similar to our previous work [46]. High quality of nanotube film has been confirmed by Raman spectrum and SEM measurements, which are free of contamination (see Figure S3). Then, SWCNT films are transferred onto the SPF by a dry-transfer technique [42, 47] and covered by index-matched liquid to enhance their interaction with light [27, 28, 36, 41]. As an index-matched overlayer, we use ionic liquid Bmim NTf_2_ with refractive index *n*_IL_ = 1.41 and no absorption at 1.55 μm wavelength. In addition, we use it to modify the absorption of the carbon nanotube film by electrochemical gating [48]. To fabricate the ionic cell, we use the same design as in previous work, see Figure 1b [47]. With no applied voltage, ionic liquid exclusively plays the role of the index-matched overlayer.

### 2.1 Light propagation simulation

First, we numerically investigate the light propagation through the SPF covered with the SWCNT film for various film thicknesses. Interband transitions require polarization along carbon nanotubes [49], which are oriented predominantly in the plane of the film. So, we are interested mostly in the polarization in the plane with a polished surface, which we further refer to as TE. We utilized COMSOL Multiphysics software to search for a stationary waveguide mode, and details of the simulation method are described in the Materials and Methods section. The parameters used for simulation are summarized in Table 1. Measurements of refractive indices of the SWCNT film and the ionic liquid overlayer are described in Materials and Methods.

**Table 1:** Parameters for simulation the light propagation through the SPF covered with the SWCNT film.

Parameter	Value
Wavelength [μm]	1.55
Core radius [μm]	4.5
Refractive index of the core	1.450
Refractive index of the cladding	1.445
Refractive index of overlayer *n*_IL_	1.41
Real part of SWCNT refractive index *n*_film_	1.43
Imaginary part of SWCNT refractive index *κ*_film_	0.62

The absorption coefficient of the SPF covered with SWCNTs depends on the imaginary part of the refractive index of the film, *κ*_film_, related to its material losses as $\alpha_{\text{film}}=\frac{4\pi }{\lambda }\kappa_{\text{film}}$ , and the overlap integral of the mode profile of the carbon nanotube film:

(1) $$\begin{array}{ll} \alpha_{\text{SPF}} & =\frac{4\pi }{\lambda }\frac{\overset{+\infty }{\underset{-\infty }{\iint }}\kappa \left(x,y\right){\left|E\left(x,y\right)\right|}^{2}dxdy}{\overset{+\infty }{\underset{-\infty }{\iint }}{\left|E\left(x,y\right)\right|}^{2}dxdy}\\ & =\frac{4\pi }{\lambda }\kappa_{\text{film}}\frac{\underset{\text{over film}}{\iint }|E\left(x,y\right){|}^{2}dxdy}{\overset{+\infty }{\underset{-\infty }{\iint }}{\left|E\left(x,y\right)\right|}^{2}dxdy}\\ & =\frac{4\pi }{\lambda }\kappa_{\text{film}}\times \text{Overlap Integral}. \end{array}$$

**Figure 2:**
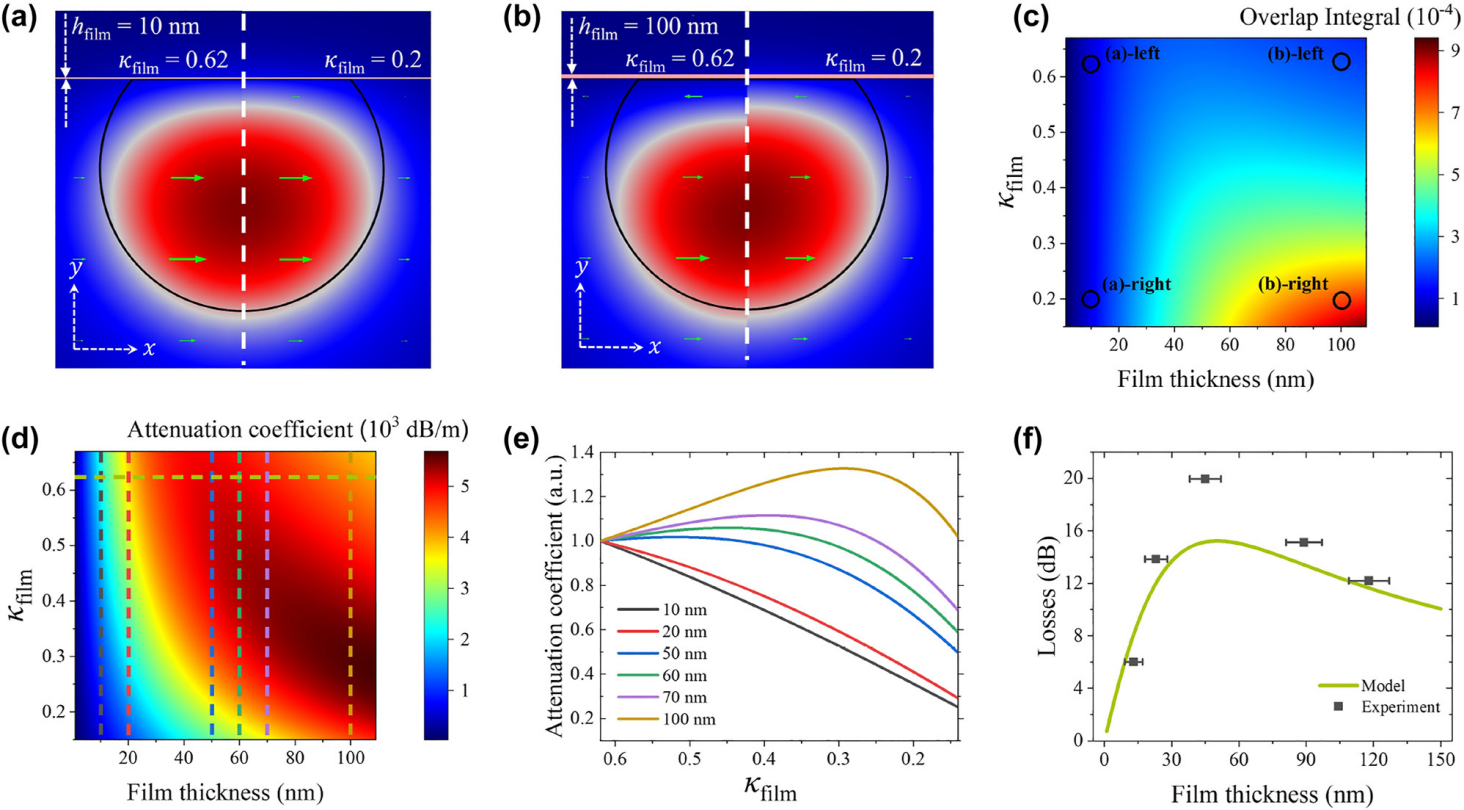
Results of the simulation of the field distribution in the SPF-SWCNT. (a and b) Cross-sectional electric field amplitude distributions of the TE modes in the SPF for selected SWCNT film thicknesses *h*_film_ = 10 and 100 nm and SWCNT film imaginary part of refractive indexes *κ*_film_ = 0.62 and 0.2. (c) Contour plot of the overlap integral of the TE mode and the SWCNT film on the SPF. (d) Contour plot of the attenuation coefficient of the SPF with covered SWCNT film. Vertical cut-lines are shown in plot (e), horizontal – in plot (f). (e) Dependence of the attenuation coefficient on *h*_film_ normalized to the initial value of *κ*_film_ = 0.62 for selected film thicknesses. (f) Modeled dependence of the attenuation on unsaturated SWCNT film thickness (line) and its experimentally measured values (dots).

Here, *λ* is the wavelength of light and we neglect the losses of the fiber and ionic liquid. We consider *κ*_film_ = 0.62 for the pristine nanotube film and model how the field distribution in SPF-SWCNT changes when *κ*_film_ value decreases. The results for relatively thin (10 nm) and thick (100 nm) films are shown in Figure 2a and b, respectively. First of all, we see that the presence of the SWCNT film deforms the mode toward the opposite side from the polished surface. For the thin film, the field profile does not change as *κ*_film_ reduces from 0.62 to 0.2. In contrast, for the thick film, the field distribution is shifted further from the surface for larger values of *κ*_film_, and moves closer, increasing the overlap integral, if *κ*_film_ is decreased. The difference can be explicitly seen in the electric field amplitude distributions of the mode along the central cut lines (Figure S6 in Supplementary Material). It leads to two competing processes: the reduction of *κ*_film_ decrease the absorption, while the increase in the overlap integral increases it. To understand how it affects the losses of the SPF-SWCNT, we plot the mode-film overlap integral Figure 2c, points with parameters corresponding to Figure 2a and b are highlighted, and attenuation coefficient *α*_SPF_ (see Figure 2d) following Equation (1) as a function of film thickness and *κ*_film_. In Figure 2c, we can clearly notice that for thin films overlap integral does not depend on *κ*_film_, while for thicker films it grows significantly for smaller *κ*_film_, which is the consequence of the mode reshaping. The resulting attenuation coefficient in Figure 2d shows monotonic behavior for smaller thicknesses. However, for thicknesses more than 50 nm, we can define two regions—where the attenuation coefficient decreases with a decrease in $\kappa_{\text{film}}\;\left(\frac{d\alpha_{\text{SPF}}}{d\kappa_{\text{film}}}>0\right)$ and opposite behavior $\left(\frac{d\alpha_{\text{SPF}}}{d\kappa_{\text{film}}}<0\right)$ separated by a point where *α*_SPF_ reaches the maximum for a given film thickness $\left(\frac{d\alpha_{\text{SPF}}}{d\kappa_{\text{film}}}=0\right)$ .

For a better visibility in Figure 2e, we demonstrate the behavior of the attenuation coefficients, normalized to initial values of *κ*_film_, for various film thicknesses corresponding to vertical cut-lines in Figure 2d. Here, we observe that for 10 and 20 nm films, the attenuation coefficient decreases almost linearly with the reduction of *κ*_film_, as expected, since the overlap integral does not change and does not contribute to the losses of the waveguide. Starting from 50 nm thick films, the slope of the attenuation coefficient curve changes the sign to positive, leading to an increase of the losses with the decrease of the *κ*_film_. Under a certain value of *κ*_film_, the absorption coefficient reaches the maximum and then starts to decrease because the growing overlap integral cannot compensate for the reduction of material losses when the *κ*_film_ gets sufficiently small. We note that this type of nonmonotonic attenuation coefficient behavior cannot be observed by variation of the real part of the refractive index of SWCNTs in the physically reachable range, see Figure S7 in Supplementary Material.

### 2.2 Experimental demonstration

As the first experimental verification, we measure small-signal losses of SWCNT films on the SPF with identical *κ*_film_ = 0.62, but different thicknesses, using the low-power CW laser (10 mW) to eliminate the contribution of nonlinear losses. According to the simulation, the SPF-SWCNT losses should have a maximum at the film thickness of 50 nm; see green line in Figure 2f. The experimental results are shown as a black point on the plot, where the film thickness was measured by the AFM. We see that in the experiment, the losses follow the same trend having the maximum for the film thickness of around 50 nm. The higher losses in the experiment we attribute to additional losses when the mode evolves from symmetric on the unpolished part of the fiber to asymmetric on polished part and back [29].

**Figure 3:**
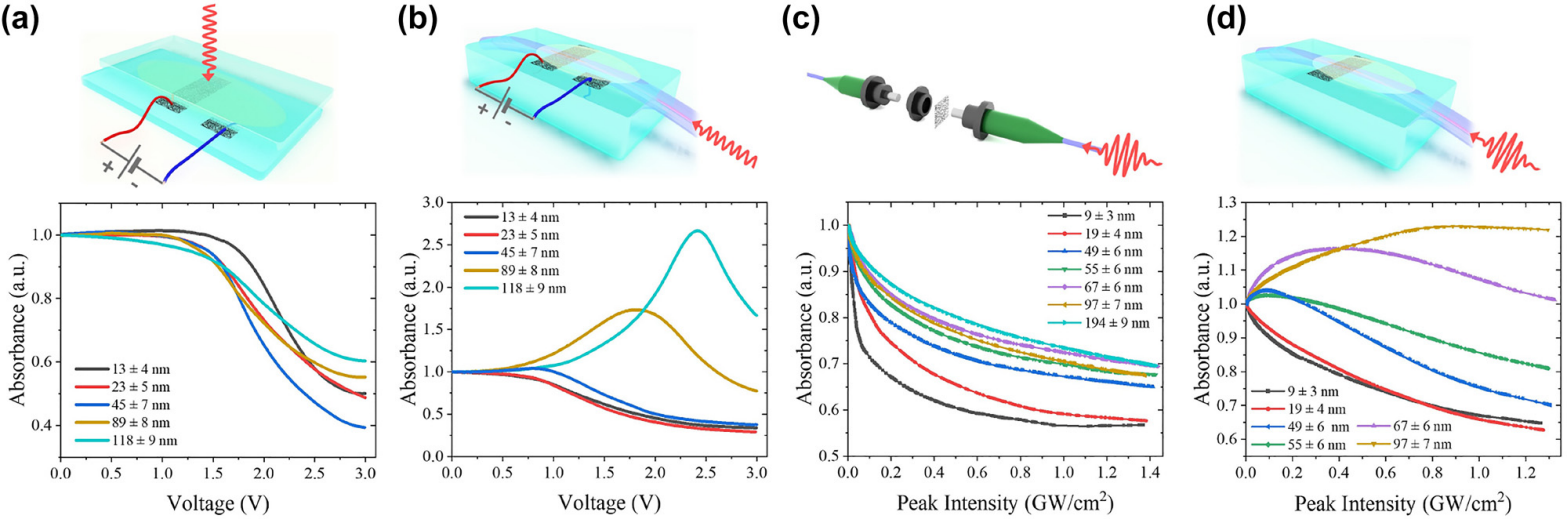
Experimental absorbance smoothed trends through the waveguide at 1.55 μm in various configurations. For better understanding, we present configurations and physics of material loss reduction in the pictures above. (a) Absorbance curves of SWCNT films of different thicknesses (values are given in the caption) on a glass substrate during electrochemical gating normalized to the nongated value. (b) Absorbance curves of the SPF covered with SWCNT films of different thicknesses during electrochemical gating normalized to the nongated value. (c) Nonlinear absorbance measurements of SWCNTs film of different thicknesses on the fiber ferrule normalized to the small-signal value (when the SWCNT film is unsaturated). (d) Measured nonlinear absorbance of the SPF covered with SWCNT films of different thicknesses normalized to the small-signal value.

Now we move to the experimental demonstration of the earlier discussed effect varying *κ*_film_. For SWCNT films, there are two possibilities to reduce the material loss coefficient: through the saturable absorption effect by the resonant excitation with ultrashort pulses [50, 51] and by electrochemical gating [52]. We start with electrochemical gating, where the application of the potential to the SWCNT film immersed in the ionic liquid leads to an electrical double-layer formation that allows to accumulate large excess charge on the surface. It causes a shift of the Fermi level toward conduction or valence band depending on the voltage sign, leading to absorption reduction [53]. We perform our measurements in two configurations, when light propagates through the gated SWCNT film from free space (whole light propagates orthogonally to the carbon nanotube film) and through SPF-SWCNT. Note that we use CW laser for this measurement with power as small as 10 mW to avoid nonlinear effects and heating. For the free-space measurements in Figure 3a, we observe that the film gets more transparent with gating regardless of thickness. The absorbance dependence of SWCNT films on the SPF for TE polarization for various film thicknesses is shown in Figure 3b. To compare it with numerical predictions in Figure 2e, all curves are normalized to nongated value and smoothed by locally weighted strategy (non-normalized graphs with data points before being smoothed can be seen in Figure S8 in Supplementary Material). Here, we see good qualitative agreement with numerically calculated results: 10 and 20 nm films demonstrate the decrease in absorbance, while for thicker films absorbance increases with applied voltage, reaches the maximum, and goes down. The difference in the curve shapes in Figures 2e and 3b comes from a functional dependence of material losses *κ*_film_ of SWCNTs on the applied voltage.

Now we investigate how the mode reshaping affects the saturable absorption, which is well known in SWCNT under resonant excitation [54, 55]. The nonlinear absorption is measured by a classic twin-detector method [47] using amplified signals from a homemade fully polarization-maintaining ultrafast fiber laser at 1.55 μm. First, we measure the nonlinear absorbance of the films deposited on the fiber connectors, where the entire mode interacts with the film in the in-plane polarization, similar to free-space measurements with gating. For all the sample thicknesses, we observe absorption saturation regardless of film thickness, see Figure 3c. In case of SPF-SWCNT samples (see Figure 3d), we observe thin films to become more transparent at high intensities while thick films demonstrate nonmonotonic behavior with increased absorption followed by absorption reduction as intensity increases, in agreement with the numerical prediction in Figure 2e. We rule out a contribution of other possible effects to the induced absorption. The two-photon absorption or other reasons of optical limiting are not observed for resonant transitions for SWCNTs. Indeed, Figure 3c evidences that the nonmonotonic behavior in Figure 3d is not an intrinsic film property but results from the geometry of light–matter interaction. To exclude thermal contribution to this behavior, we check that there is no nonlinear absorbance for the thickest SWCNT film on the SPF if a continuous wave laser with the same average power is used for nonlinear absorbance measurements (see Figure S11a in Supplementary Material). Finally, we check that polarization rotation does not take place for SPF-SWCNT if the polarization of the light coincides on the entrance with slow or fast axis, see Figure S11b.

Thus, regardless of how the material losses of SWCNTs are changed experimentally, the examined effect of the mode reshaping in the SPF with thick SWCNT films can be detected for the TE polarization. We do not observe the same effect for TM polarization, which is also in agreement with numerical simulation. The results of the measurements and numerical model for out-of-plane polarization can be found in Supplementary Material S5.

**Figure 4:**
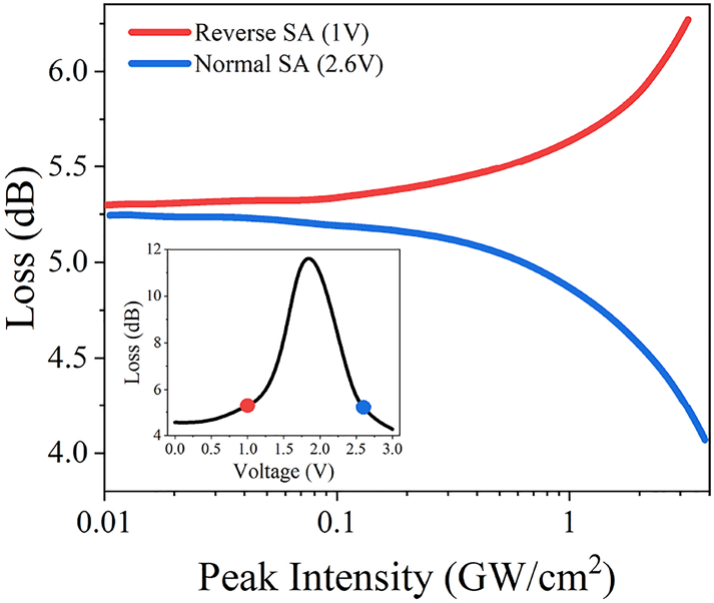
Nonlinear losses measurements of SPF-SWCNT of 107 ± 9 nm film thickness under gate of 1 V and 2.6 V (curves are smoothed) and inserted graph of its small-signal loss dependence during electrochemical gating (nonlinear loss measurement points are highlighted).

Now we combine the two mentioned aforementioned approaches to demonstrate how nonlinear absorption can be switched between saturable and induced absorption in a single device in a reproducible manner. Previous investigation of ionic liquid gated SWCNT film on SPF has demonstrated that applied voltage leads to a decrease of the modulation depth of the saturable absorption [42]. In that study, the experimental conditions corresponded to what we call here a thin film case where small signal losses are also decreasing under voltage. Now let us consider a thick film, where small signal absorption shows nonmonotonic behavior on voltage. Let us choose two points on the opposite slopes of the curve that correspond to nearly the same small signal losses (see insertion in Figure 4) and measure nonlinear absorption there. The results are shown on Figure 4. For the point on ascending loss slope (1 V gating voltage), reverse saturable absorption is observed, while on the opposite slope (2.6 V gating voltage), we see normal saturable absorption. This approach demonstrates reconfigurable nonlinear loss engineering where small signal losses are kept identical. Nonlinear transmittance measurements for these (before being smoothed) and other voltages are represented in Figure S12 for the sake of completeness.

### 2.3 Analytical approach

**Figure 5:**
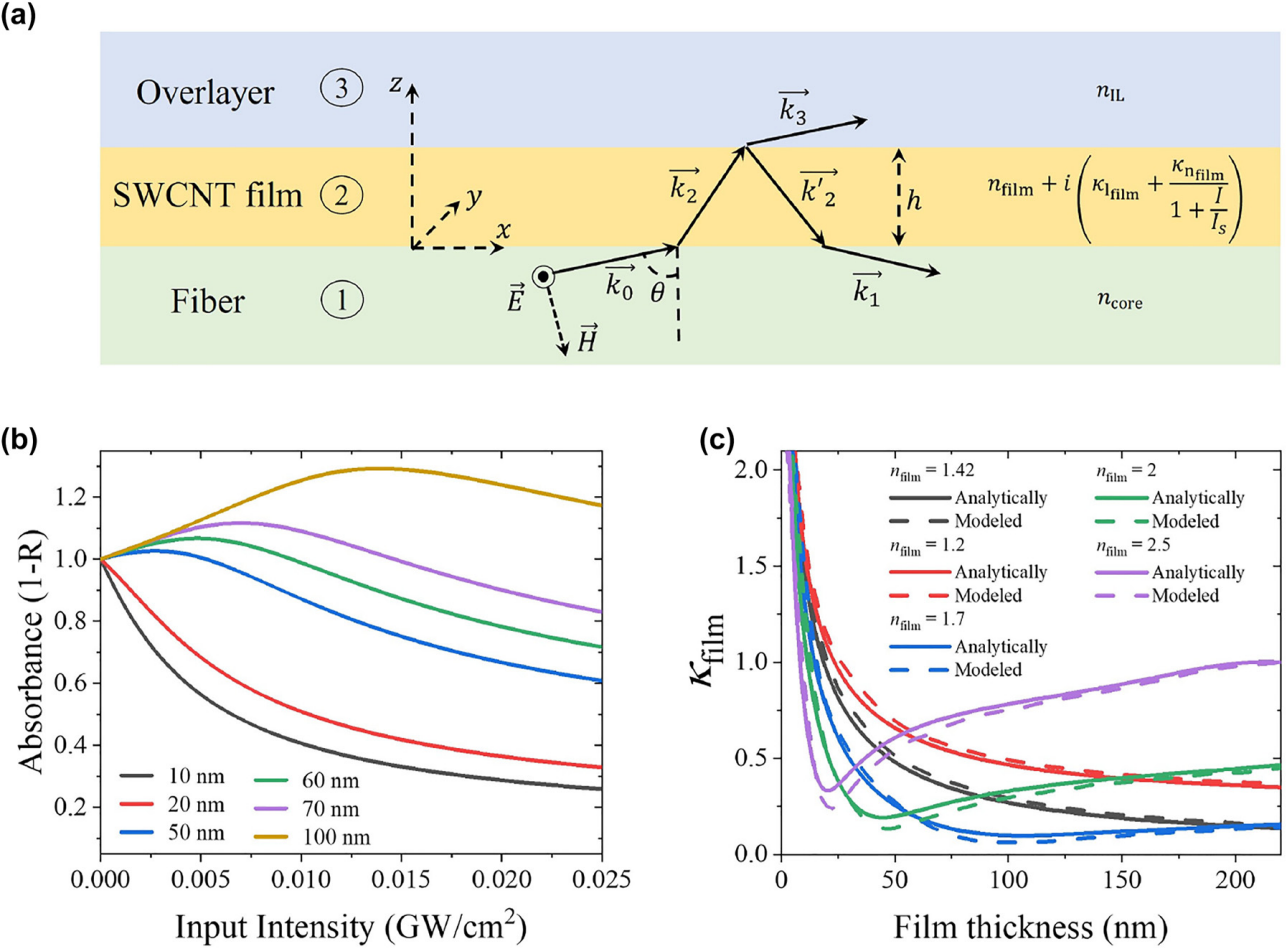
Analytical consideration of absorbing material covered waveguides. (a) Sketch of virtual experiment for analytical approach: plane wave from media (1) incident under acute angle on the thin saturable absorber film (2) undergoes the total internal reflection from media (3). (b) Nonlinear absorbance, calculated as 1 – reflection, from SWCNT films of different thicknesses normalized to the small-signal value. (c) The curves, defined by condition $\left(\frac{d\alpha_{\text{SPF}}\left(n,d\right)}{d\kappa_{\text{film}}}=0\right)$ , which divide the film parameter space in normal and reverse saturable absorption for different real parts of the SWCNT refractive index.

The effect of switching saturable absorption to reverse effect can be resembled analytically if we consider a straightforward case of the plane wave reflected by the saturable absorber film under the total internal reflection condition. The scheme of the virtual experiment is shown in Figure 5a. We consider a plane wave incident under an acute angle from an optically dense media to a thin layer with a saturable absorption followed by overlayer. To compare analysis with experimental results, we use the refractive indices of layers corresponding to the fiber core, carbon nanotubes, and ionic liquid (see Table 1). We use an approach similar to Landau et al. [56], where we consider the intermediate layer’s saturable absorption. The details of the calculations are shown in Supplementary Material S6. Figure 5b presents the dependences of the absorption for various SWCNT film thicknesses for the angle of incidence equal to 85°, which meets total internal reflection angle requirements of the optical fiber. The results are very similar to experimental observations of the nonlinear response of the SPF-SWCNT in Figure 3d: absorption goes down with intensity for the small thickness while for 50 nm and higher absorption shows a gradual increase followed by absorption reduction. This effect preserves all the angles of wave incidence for given refractive indices if the total reflection condition is fulfilled.

Finally, we would like to define a range of parameters of the film, where reverse saturable absorption can be observed. The regions with normal absorption and reverse effect are separated by the condition $\left(\frac{d\alpha_{\text{SPF}}\left(n,d\right)}{d\kappa_{\text{film}}}=0\right)$ , where absorption is maximum for a given film thickness and refractive index is reached. In Figure 5c, we plot curves satisfying this condition in coordinates of film thickness and *κ*_film_ for various values of the real part refractive index of the film by using the analytical approach (solid lines) and data from Figure 2d (dashed lines). Below the curve, a normal saturable absorption is expected, while above the curve, a reversible effect should be observed. We find analytical curves and modeling results in remarkable agreement despite the differences in the modes of cylindrical waveguide and plane waves. The correspondence between the analytical approach and modeling is shown more explicitly in Figure S16b in Supplementary Material for *n*_film_ = 1.42. The largest deviations between theory and modeling are observed near the dips for the curves with larger values of the refractive index (*n*_film_ = 1.7, 2, 2.5). These points correspond to the phase-matching condition of the modes in the fiber and in the film, leading to a situation where a significant part of the mode propagates within the film. These parameter regions cannot be handled properly by our simple analytical approach and mode propagation through coupled waveguides should be considered here.

The effect under consideration is general for waveguiding systems, covered by a thin absorbing film, including waveguides of the rectangular profile used in many applications from semiconductor lasers to integrated optics. It can also work in a reverse manner if the waveguide is covered by the material with optical limiting. In this case, the nonlinear increase of absorbance of covering material will turn into a saturable absorption for waveguide propagating light for a properly tuned film thickness and refractive index. It opens the perspectives for engineering of the nonlinear optical response, which can be implemented in all-optical computing and neuromorphic integrated photonic systems [57].

## 3 Conclusions

In summary, using carbon nanotube thin films deposited on a side-polished fiber, we have demonstrated that the light mode in the waveguide experiencing evanescent-field interaction with the covering material can be modified by changing the material losses of the covering film. It can lead to several practical effects like nonmonotonic dependence of waveguide losses on the overlaying film thickness and, more remarkably, turning of the saturable absorption in photoinduced absorption. Moreover, it allows us to assemble an optical device with reconfigurable nonliner losses and demonstrate reproducible controllable switching between normal and reversed saturable absorption. We have shown that this effect is strongly dependent on the thickness of the film and can be observed if the thickness exceeds a certain value. Our analytical findings suggest that this effect is not limited to a side polished fiber configuration but can be observed in a wide range of waveguide systems covered by the absorbing material once requirements on refractive index and covering film thickness are met.

## 4 Materials and methods

### 4.1 SWCNT film synthesis and transfer onto the SPF

Single-walled carbon nanotube synthesis method employs aerosol CVD based on the Boudouard reaction with ferrocene acting as a catalyst precursor and CO as a carbon source described elsewhere [58]. A flow of carbon monoxide CO and Fe-based nanoparticles heated to 880 °C in a tubular furnace reactor resulted in the formation of SWCNTs [59]. The films of the SWCNTs are collected downstream of the reactor on a nitrocellulose filter. The film is a mixture of semiconducting and metallic SWCNTs that form a random network with a predominant orientation in the film surface. The mean diameter of SWCNTs can be controlled during the synthesis process in the range from 1.0 to 2.3 nm by introducing a certain amount of carbon dioxide (CO_2_) in the reactor [45, 60]. The thickness of the SWCNT film on a filter can be controlled with high precision by the collection time. We use a commercial side-polished fiber (KS Photonics inc). SWCNT films are transferred onto the SPF by the dry-transfer technique: collected on the nitrocellulose filter SWCNT films are pressed toward the polished fiber surface. Due to a weak adhesion to the filter and the high specific surface area of the SWCNTs, films can be easily dry transferred to practically any other substrates by simply pressing it down [42]. For the saturable absorption experiments, the length of the film was chosen so that the small signal (unsaturated) loss through the fiber was around 3 dB to avoid effects of detector nonlinearity and significant intensity reduction along the covered part. For the densification, the SWCNT films were first soaked in a drop of ethanol and subsequently dried out. To fabricate an ionic cell, we used the same design as in previous work [47], so another SWCNT film was transferred in the immediate vicinity for a counter electrode that does not cover the polished part and thus is not affecting the losses. The electrode wires are fixed to the block with a silver paste. Then, the ionic liquid (Bmim NTf_2_) is dripped on the sample to cover both electrodes. Ionic liquid was synthesized by the standard procedure including alkylation 1-methylimidazole by 1-bromobutane and following anion exchange with lithium bistriflimide in the water phase [61].

### 4.2 SWCNT films and ionic liquid constant measurements

The complex refractive indices of the SWCNT film and ionic liquid are measured at 1.55 μm. The imaginary part of the refractive index of SWCNTs is retrieved from fitting absorbance of nanotube films of different thicknesses deposited on fiber ferrules: *κ*_film_ = 0.62 ± 0.06 (Figure S4 in Supplementary Material). We measure the real part of the refractive index by measuring the reflected power from the fiber end with a deposited SWCNT film thick enough to absorb all the light so reflection from the second interface can be neglected. Similarly, the refractive index of ionic liquid was measured by dripping fiber end into the liquid (see Figure S5). The refractive index for carbon nanotubes, calculated using Fresnel equation and known refractive index of the fiber core, varies between 1.35 and 1.6 and were chosen as *n*_film_ = 1.43 to provide best correspondence with experimental results. A large range of its values is attributed to a variation in nanotube density during liquid densification and transfer onto the fiber ferrule. The refractive index of the ionic liquid is *n*_IL_ = 1.41 ± 0.01 in good agreement with the extrapolated values from the literature [62–64]. As a reference, we measure the refractive index of water: *n*_water_ = 1.319. The deviation from the known value (*n*_water_ = 1.318 [65]) is in the third decimal place, which confirms the high accuracy of the method.

### 4.3 Electrochemical gating and nonlinear transmittance measurements

As a light source for measurements with electrochemical gating, we use Pure Photonics PPCL550 tunable laser at 1.55 μm with narrow 10 kHz linewidth and ultra-low FM noise. In the case of free-space measurements, an ionic cell with two SWCNT film electrodes on a glass substrate is prepared using SWCNT dry transfer technique. We utilize Potentiostat R-45X with module for measuring electrochemical impedance FRA-24 M by Electro Chemical Instruments to apply voltage and monitor electrochemical characteristics of gated SWCNTs. Light power is monitored using the power meter Thorlabs PM100D.

For nonlinear optical absorption measurements, we use a homemade fully polarization-maintaining ultrafast fiber laser at 1.55 μm as a source of pulses, which is mode-locked by the SWCNT film on the fiber ferrules. The commercial erbium-doped fiber amplifier Keopsys PEFA-SP-C-PM-27-B130-FA-FA is employed to amplify the signal from the laser. Then, the light is being attenuated and split by a high-power coupler. One output of the coupler works as a reference and another one is used for exposure of the sample. We monitor the light power using the dual-channel optical power meter Thorlabs PM320E. The scheme of the described experimental setup can be seen in Figure S9 in Supplementary Material. Measured pulse autocorrelation and optical spectrum can be found in Figure S10.

### 4.4 Light propagation simulation method

The electromagnetic field distribution in cross section inside in the SPF covered by the SWCNT film is modeled using COMSOL Multiphysics software, Wave Optics module, and mode analysis in frequency domain by searching the fundamental mode around the effective mode index 1.4474. Perfectly matched layers are used on the borders of the computation window.

## Supplementary Material

Supplementary Material Details
