# Supplementary material for: Reconfigurable nonlinear losses of nanomaterial covered waveguides
Source: Nanophotonics. 2023 Oct 23;12(22):4229–38. doi: 10.1515/nanoph-2023-0563 (PMC11501370; doi:10.1515/nanoph-2023-0563)
Supplement: Supplementary file 1 — Supplementary Material Details [file j_nanoph-2023-0563_suppl_001.pdf]

Supplemental Material for

Reconfigurable nonlinear losses of nanomaterial covered waveguides

Ayvaz Davletkhanov [1], Aram Mkrtchyan[1], Alexey Bunkov[1], Dmitry Chermoshentsev[1,2], Mikhail Shashkov[2], Daniil Ilatovskii[1], Dmitry Krasnikov[1], Albert Nasibulin[1], Yuriy Gladush[1]

[1]*Skolkovo Institute of Science and Technology, Moscow 143026, Russia*

[2]*Russian Quantum Center, Skolkovo, Moscow, 121205, Russia*

[3]*Boreskov Institute of Catalysis SB RAS, Novosibirsk 630090, Russia*

S1. SWCNT film synthesis and transfer onto the SPF.

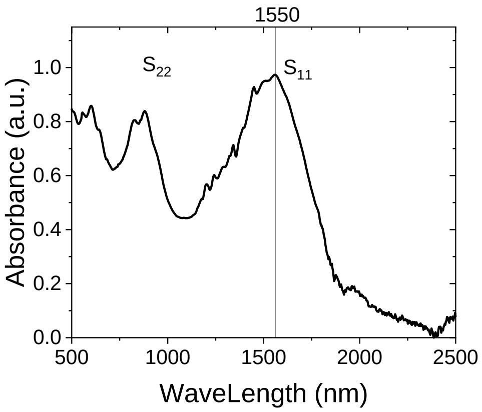

Figure S1. Measured absorbance spectrum of SWCNTs with S_{11} around 1550 nm.

S2. SWCNT films and ionic liquid constants measurements.

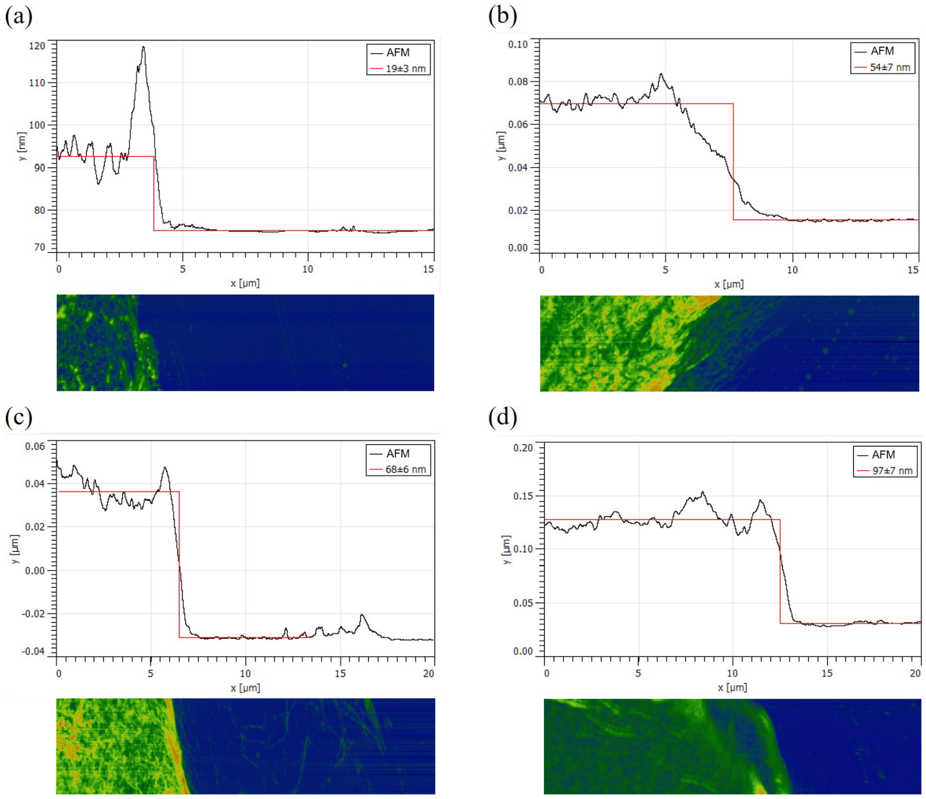

Figure S2. AFM topographies of SWCNT films deposited on a silicon substrate and corresponding height distributions averaged over 5 μm along vertical direction for different thicknesses.

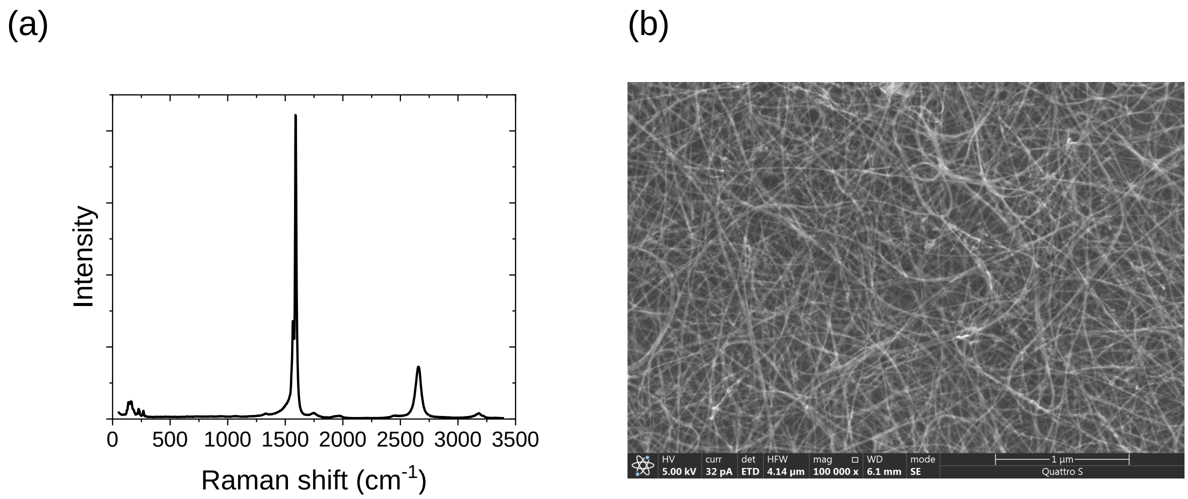

Figure S3. (a) Raman spectrum and (b) SEM image of SWCNT films deposited on a silicon dioxide substrate following the same techniques as described in the Material and Methods section.

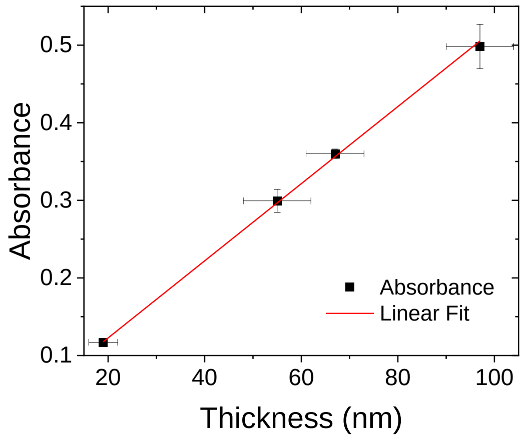

Figure S4. Measured small-signal (unsaturated) losses of SWCNT films of different thicknesses on the fiber ferrules at 1.55 µm.

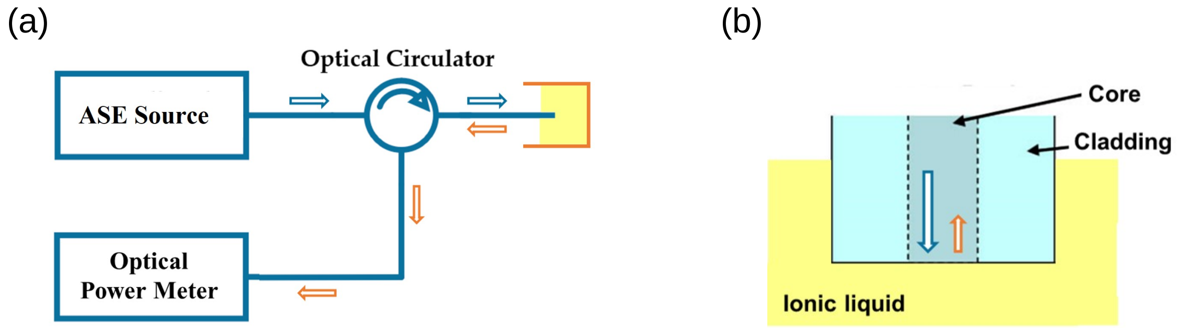

Figure S5. (a) The experimental setup for the refractive index measurement. (b) The fiber end in the ionic liquid/SWCNTs.

S3. Modeling in COMSOL.

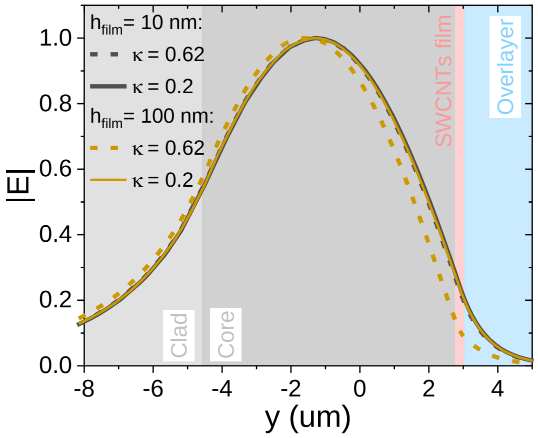

Figure S6. Normalized electric field envelope distribution of the mode along the central cut-lines in the SPF-SWCNT for selected film thicknesses h_{film} = 10 and 100 nm and SWCNT film imaginary part of refractive indices κ_{film} = 0.62 and 0.2. It shows that for the thickness of 100 nm and unchanged κ_{film} the mode is shifted from the film, that causes a decrease in overlap integral. The reduction of κ_{film} attracts the mode to film, consequently, the overlap integral rises.

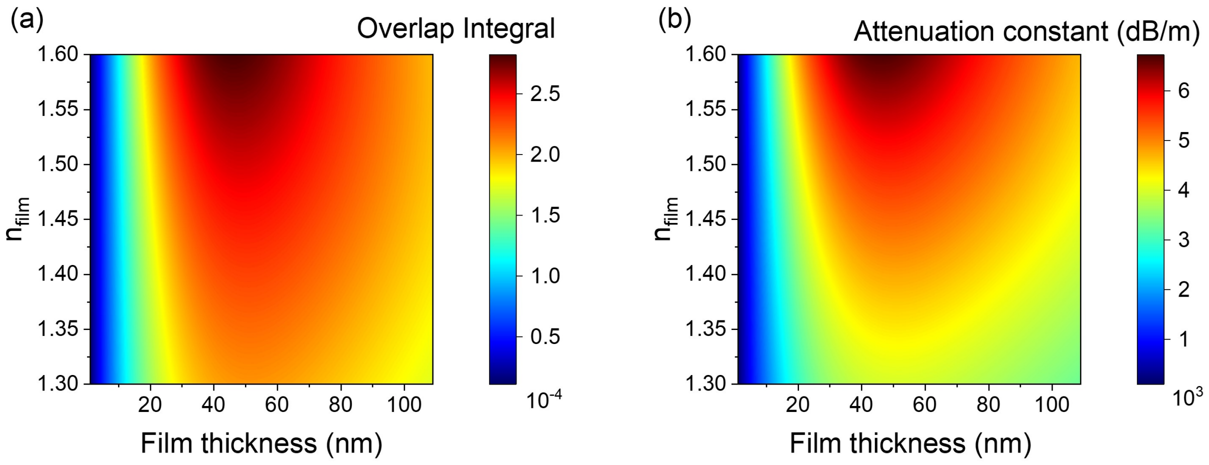

Figure S7. Variation of the real part of the refractive index and thickness of SWCNT film on the SPF. (a) Contour plot of the overlap integral of the mode and the SWCNT film. (b) Contour plot of the attenuation constant. It proves that the phenomenon under discussion cannot be attributed to a change in the real part of the refractive index of SWCNT film.

S4. Nonlinear absorbance measurements.

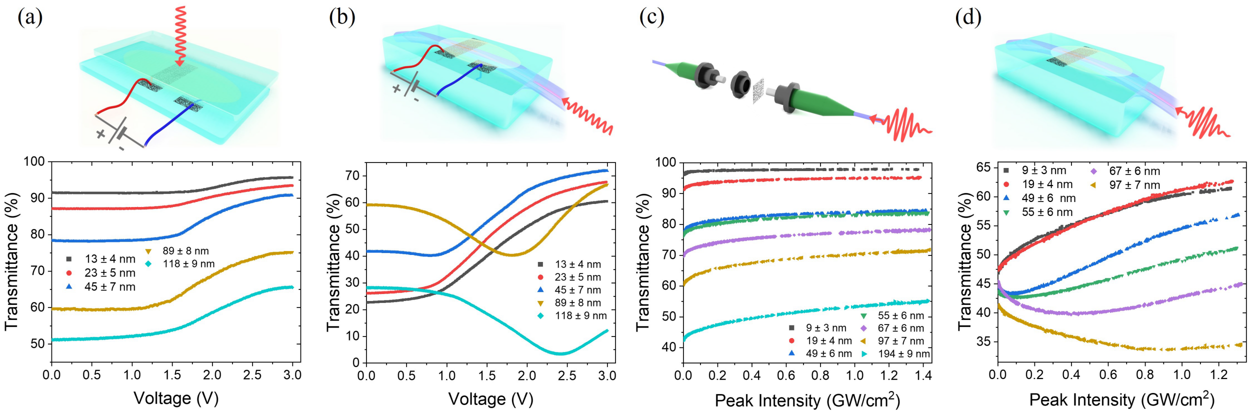

Figure S8. (a) Transmittance curves of SWCNT films of different thicknesses (values are given in the caption) on a glass substrate during electrochemical gating. (b) Transmittance curves through the SPF with SWCNT films of different thicknesses during electrochemical gating. (c) Nonlinear transmittance measurements of SWCNTs film of different thicknesses on the fiber ferrule. (d) Measured nonlinear transmittance through the SPF covered with SWCNT films of different thicknesses.

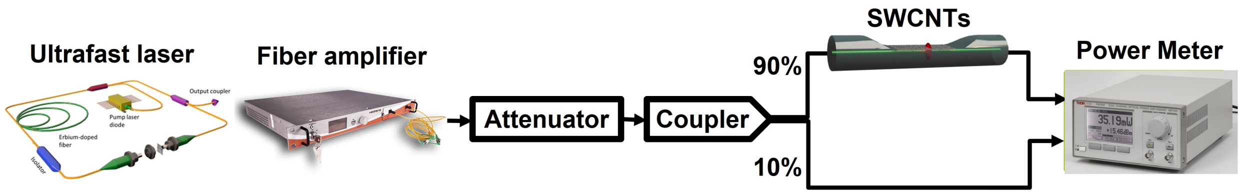

Figure S9. Scheme of nonlinear optical absorption measurement by a classic twin-detector method using amplified pulses from ultrafast fiber laser[1].

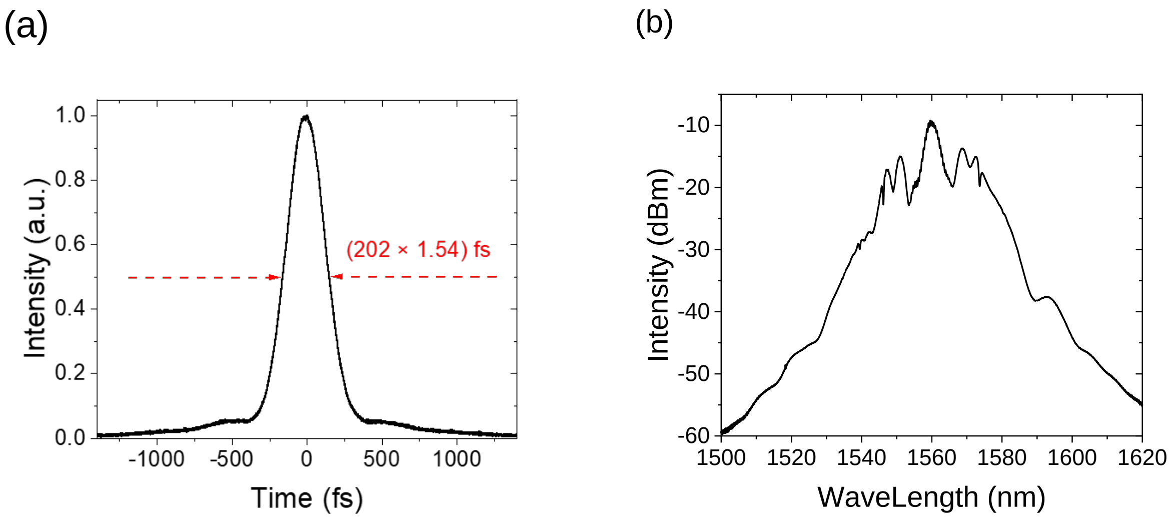

Figure S10. Characteristics of pulses used for nonlinear absorption measurements. (a) Measured pulse autocorrelation function after amplification and (b) its optical spectrum.

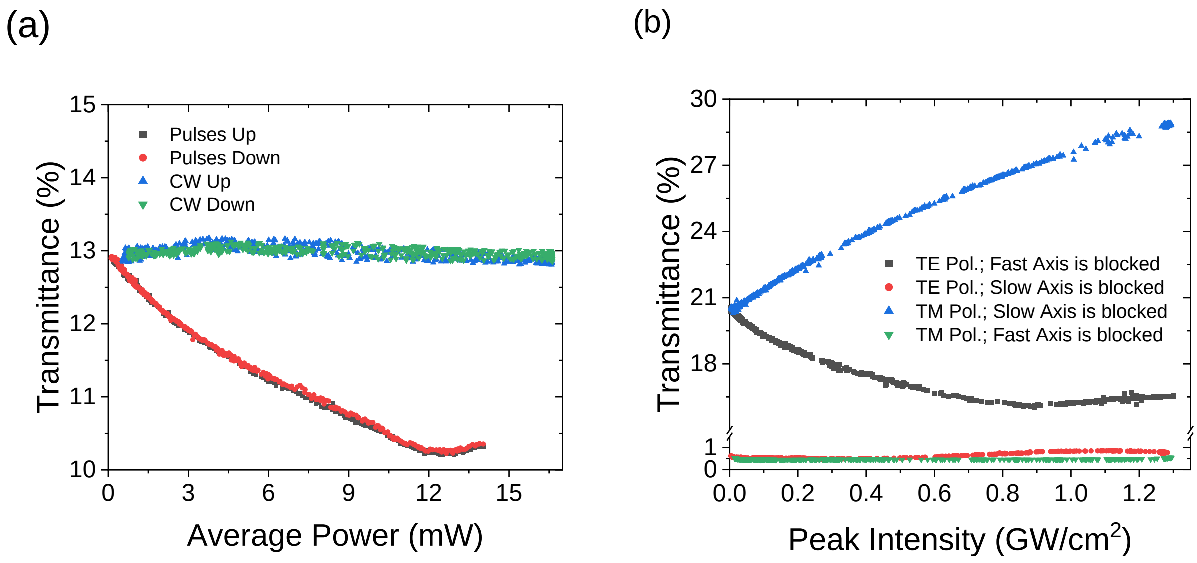

Figure S11. (a) Measured nonlinear transmittance of the SPF covered with the SWCNT film of (97 ± 7) nm thickness on it using amplified pulses or continuous wave (CW) signal from low to high power values (Up) and versa verse (Down). It proves that we cannot observe the considered phenomenon using the CW signal of the same power, and therefore we can exclude thermal contribution to this behavior. (b) Measured nonlinear transmittance through the SPF-SWCNT of (97 ± 7) nm film thickness blocking the slow or fast axis of the fiber after the SPF during measurements. It demonstrates that there is no nonlinear polarization rotation that can provide a considerable contribution to the effect.

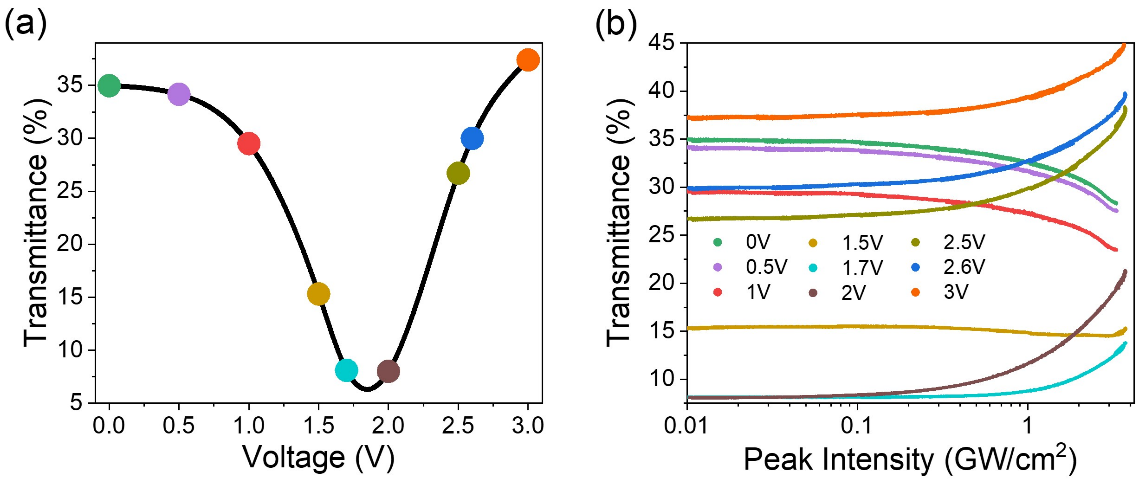

Figure S12. (a) Small-signal transmittance dependence during electrochemical gating of SPF-SWCNT of 107 ± 9 nm film thickness and (b) its nonlinear transmittance measurements for highlighted voltage points.

S5. TM polarization

The examined phenomenon takes place when the light in the SPF is polarized in the plane of the film. This is a common thing to work in in-plane polarization, since nanotubes are predominately oriented parallel to the film and, consequently, the probability of absorbing a photon with the electron interband transition is greater [2]. Indeed, the measured nonlinear absorbance of thin films (19 ± 4) nm when the light in the SPF is polarized perpendicularly is considerably smaller than in in-plane polarization, which can be seen in Figure S13. In addition, with an increase in film thicknesses to (97 ± 7) nm the sign of the nonlinear absorbance is not changed, on the contrary, the modulation depth of the saturable absorbance is enlarged. By modeling the TM mode in the SPF, it becomes clear that we cannot see the discussed in the main manuscript effect of the mode modification in this case (Figure S14). The enhancement of the saturable absorbance effect for thicker films is attributed to an increase in the overlap integral that increases the interaction strength, and therefore the nonlinear effect.

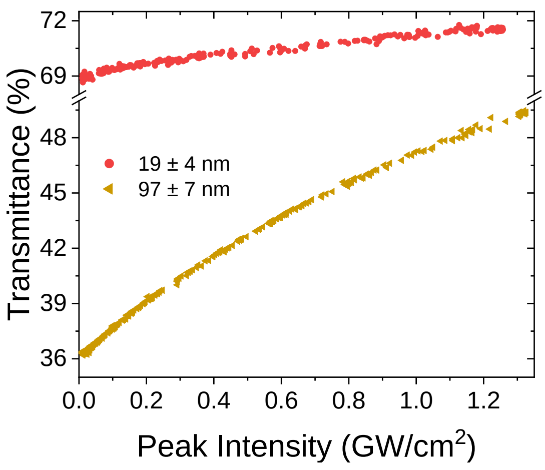

Figure S13. Nonlinear transmittance measurements of the SPF-SWCNT of different thicknesses (values are given in the caption) in case of TM polarization.

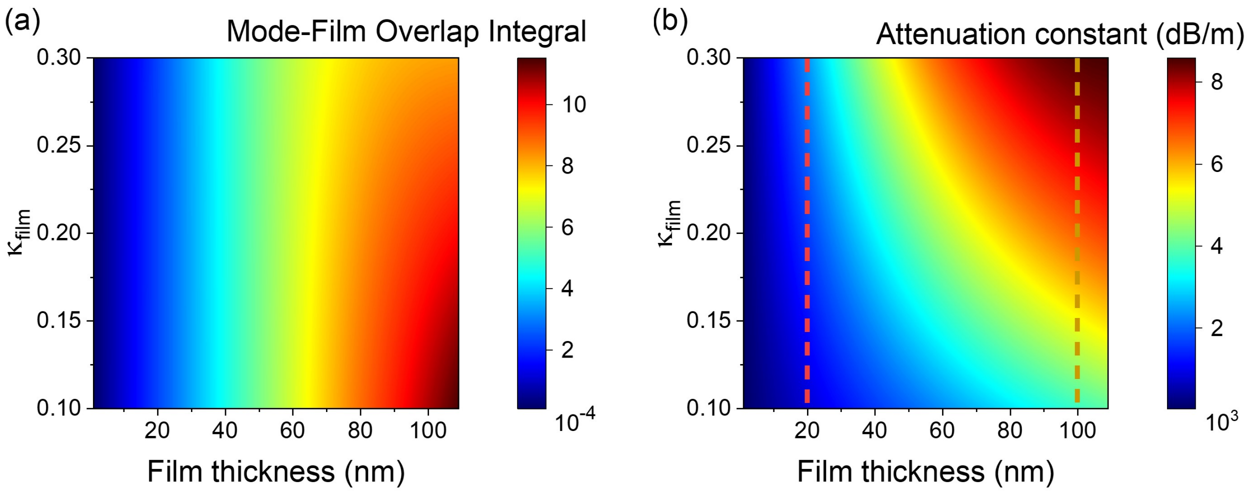

Figure S14. Modeled light propagation through the SPF-SWCNT in case of TM polarization. (a) Contour plot of the overlap integral of the mode and the covering SWCNT film. (b) Contour plot of its attenuation constant for SPF-SWCNT.

S6. Analytics

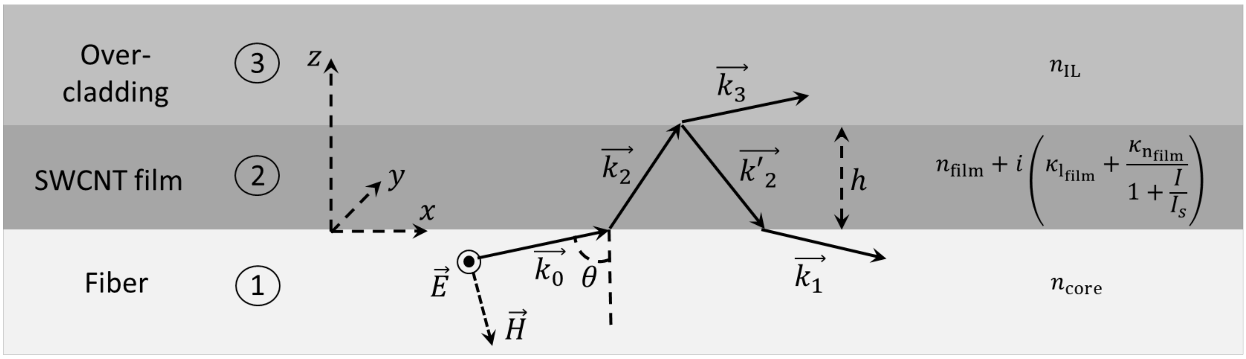

Figure S15. Sketch of virtual experiment for analytical approach: plane wave from media 1 incident under acute angle on the thin saturable absorber film (2) undergoes the total internal reflection from media 3.

Here, we consider the reflection, refraction, and absorbance of a monochromatic plane wave incident on a flat absorbing layer between two homogeneous transparent media. The light comes from medium one, corresponding to the fiber core followed by the SWCNT film (absorbing media 2), then, after refraction, the wave approaches the second interface with the over cladding (media 3). We will define the values related to the incident, reflected, and refracted waves at the first interface, respectively, with indices 0, 1, and 2. Reflected and refracted waves at the second interface are notated with indices 2' and 3, respectively (Figure S15). Due to the uniformity in the *x* direction, the dependence of the solution of the field equations on this coordinate in the entire space should preserve:

$$k_{0x} = k_{1x} = k_{2x} = k_{3x} = \frac{2\pi}{\lambda} n_1 \sin\theta.$$

It gives us the following relations:

$$k_{1z} = -k_{0z} = -\frac{2\pi}{\lambda}\ n_1 \cos\theta,$$

$$k_{2z} = -k'_{2z} = \sqrt{k_2^2 - k_{2x}^2} = \frac{2\pi}{\lambda}\sqrt{\left(n_2 + \mathrm{i}\left(\kappa_l + \frac{\kappa_n}{1+\frac{I}{I_s}}\right)\right)^2 - n_1^2 \sin^2\theta},$$

$$k_{3z} = \sqrt{k_3^2 - k_{3x}^2} = \frac{2\pi}{\lambda}\sqrt{{n_3}^2 - n_1^2 \sin^2\theta},$$

where

$$I = \frac{1}{2} c\epsilon_0 |A_2 + A'_2|^2.$$

Here, A_2, and A_2' are the fields on the first interface in media 2 after refraction and after reflection from media 3 respectively. We neglect the reduction of intensity across the second layer for calculating the nonlinear contribution to the refractive index. This approximation is valid for the thin layers when absorption is not very high, see Figure S16(a).

The boundary conditions for S polarization impose continuity of $\mathrm{A} = \mathrm{E_y}$ and $\mathrm{H_x} = -\mathrm{ck_zE_y}/\omega$, thus:

Interface 1 – 2:

$$A_0 + A_1 = A_2 + A_2',$$

$$k_{0z}(A_0 - A_1) = k_{2z}(A_2 - A_2');$$

Interface 2 – 3:

$$A_2 e^{i\psi} + A_2' e^{-i\psi} = A_3,$$

$$k_{2z}\left(A_2 e^{i\psi} - A_2' e^{-i\psi}\right) = k_{3z} A_3.$$

Here exponential terms take into account the phase change and absorption of the wave in the second layer of the thickness *h:*

$$\psi = k_{2z} h = \frac{2\pi}{\lambda} h \sqrt{\left(n_2 + i\left(\kappa_l + \frac{\kappa_n}{1+\frac{I}{I_S}}\right)\right)^2 - n_1^2 \sin^2\theta}.$$

From the boundary conditions, we find the dependence of reflected wave A_1 on the incident wave A_0:

$$\frac{A_1}{A_0} = \frac{(k_{0z} + k_{2z})(k_{2z} - k_{3z})e^{2i\psi} + (k_{0z} - k_{2z})(k_{2z} + k_{3z})}{(k_{0z} - k_{2z})(k_{2z} - k_{3z})e^{2i\psi} + (k_{0z} + k_{2z})(k_{2z} + k_{3z})}$$

Without nonlinearity it provides the explicit formula for reflection:

$$R = \left|\frac{A_1}{A_0}\right|^2.$$

If we take into account saturable absorption, the parameters k_{2z}, ψ are becoming functions of A_1, A_0 as $I \sim |A_2 + A_2'|^2 = |A_1 + A_0|^2$. It gives the implicit equation for A_1, from which we numerically calculate the absorbed light under total reflection condition as $A = (1 - R)$, see Figure 5(b) in the main text.

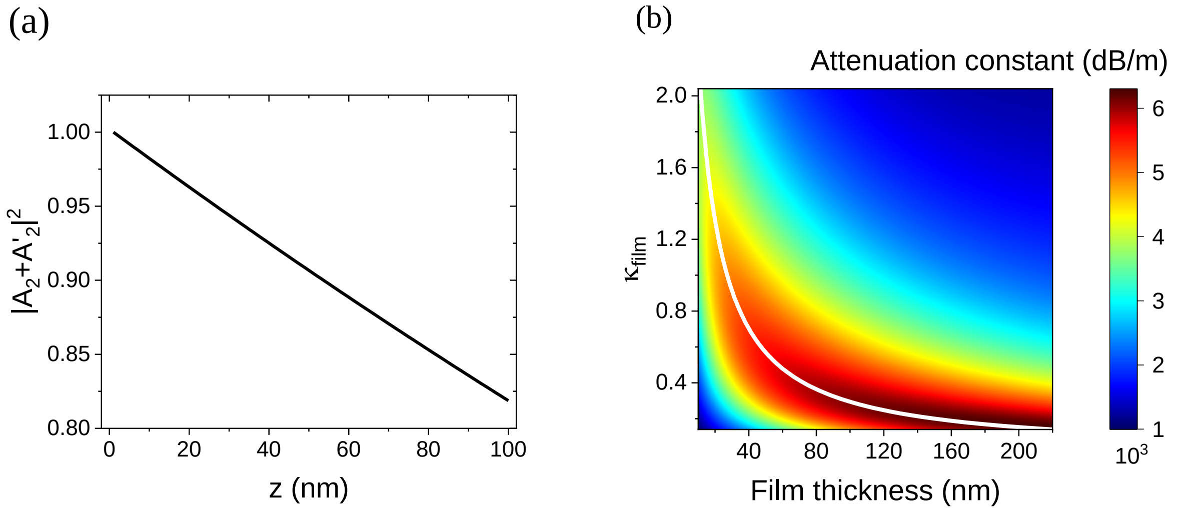

Figure S16. (a) Dependence of the field amplitude along the wave propagation through the film normalized to the total field immediately after fiber/film interface, the angle of incidence is 85°. (b) Comparison of the modeled attenuation constant of the SWCNT-SPF and the analytically obtained curved (white curve) for condition $\frac{d\alpha_{SPF}(n,d)}{d\kappa_{film}} = 0$ for n_{film}=1.425. The perfect agreement can be noticed.

Bibliography

(1) Gladush, Y.; Mkrtchyan, A. A.; Kopylova, D. S.; Ivanenko, A.; Nyushkov, B.; Kobtsev, S.; Kokhanovskiy, A.; Khegai, A.; Melkumov, M.; Burdanova, M.; Staniforth, M.; Lloyd-Hughes, J.; Nasibulin, A. G. Ionic Liquid Gated Carbon Nanotube Saturable Absorber for Switchable Pulse Generation. *Nano Lett.* **2019**, *19* (9), 5836–5843. https://doi.org/10.1021/acs.nanolett.9b01012.

(2) Ko, J.; Jeong, H.; Choi, S. Y.; Rotermund, F.; Yeom, D. Il; Kim, B. Y. Single-Walled Carbon Nanotubes on Side Polished Fiber as a Universal Saturable Absorber for Various Laser Output States. *Curr. Appl. Phys.* **2017**, *17* (1), 37–40. https://doi.org/10.1016/j.cap.2016.10.019.
